# Exploring How Staff Engage in Conversations Around Sexuality With Adults With Intellectual Disabilities: A Qualitative Systematic Review

**DOI:** 10.1111/jar.70319

**Published:** 2026-10-20

**Authors:** Naoise Webb, Hannah O'Daly, Christine Linehan

**Affiliations:** ^1^ University College Dublin Dublin Ireland

## Abstract

**Aims:**

This review explored how health and social care staff engage in sexuality‐related conversations with adults with intellectual disabilities.

**Method:**

Electronic databases (PsycINFO, MEDLINE, CINAHL and Scopus) were searched from January 2006 to August 2025 following PRISMA guidelines. From the initial 1666 studies identified, a total of 23 were retained for narrative synthesis following quality assessment.

**Results:**

A total of 75 sexuality‐related conversations were identified, of which 42 reflected real interactions (occurring [*n* = 39] or actively avoided [*n* = 3]) and 33 were hypothetical. Facilitators of conversations included trusting relationships, supportive organisational structures and targeted staff education. Key barriers included fear, stigma, role ambiguity and risk‐averse service cultures. Findings highlighted a tension between growing recognition of sexual rights and risk‐driven practice, underscoring the need for staff training, role clarity and rights‐based policies to support open dialogue.

**Trial Registration:**

PROSPERO: CRD42023454984

## Lay Summary

- People with intellectual disabilities have the same sexual feelings and needs as everyone else, but often do not get the support they need.
- Talking about sex and relationships is important, but this research suggests that conversations rarely happen. We explored why that may be and found that staff often avoid these conversations because they feel unsure, worried or embarrassed.
- When these conversations do happen, they work best when there is trust between the person and their support worker, and when staff feel confident and supported in their role.
- Things need to change. Staff need better training, and organisations need clearer rules that put the rights and wishes of people with intellectual disabilities first—not just what others think is best for them.

## 1 Introduction

Sexuality is a fundamental aspect of human experience and an important component of identity, well‐being, relationships and quality of life. Extending across the lifespan, sexuality encompasses sex, gender identity, sexual orientation, eroticism, pleasure, intimacy and reproduction, and is shaped by the complex interplay of individual, relational, social and cultural experiences (World Health Organization [WHO] 2006). Despite its centrality to human life, sexuality remains an area in which individuals with intellectual disabilities can experience significant barriers to expression, recognition and support.

There is a growing understanding around the importance of sexuality‐related discourse in order to support individuals with intellectual disabilities to construct a healthy sexual identity, fulfil their sexual needs and develop skills to navigate sexual situations safely (Correa et al. 2022). Conversations are an essential step in supporting adults with intellectual disabilities to protect their sexual boundaries and develop assertiveness (Svae et al. 2022). Research indicates that individuals with intellectual disabilities express similar sexual desires and needs as the general population (Borawska‐Charko et al. 2017), but these needs are often unmet or dismissed (Borawska‐Charko et al. 2017; Carter et al. 2022; Fish and Björnsdóttir 2022; Neuman 2022). In this study, the term ‘support staff’ is used as an umbrella term to refer to the diverse range of health and social care professionals (HSCP), including direct support workers, clinicians, social care workers and educators, who provide support to adults with intellectual disabilities across residential, community and clinical settings. This broad definition reflects the varied terminology used across the literature and enables a comprehensive consideration of the professionals involved in supporting adults with intellectual disabilities.

Adults with intellectual disabilities have historically been excluded from sexuality discourse, reinforcing stigma and limiting their dignity, autonomy and sexual rights (Schalock et al. 2021; McConnell and Phelan 2022). Prevailing assumptions that they are asexual, vulnerable or childlike have contributed to paternalistic and risk‐oriented practices, in which protection from harm may be prioritised over autonomy, intimacy and opportunities for sexual expression (Fegan et al. 1993; Gill 2015; Charitou et al. 2021). Such approaches can restrict access to comprehensive sexuality education, relationships and opportunities to develop knowledge and skills relating to sexual decision‐making. However, there is growing recognition that adults with intellectual disabilities have the same rights to sexual health, relationships and sexual expression as their non‐disabled peers (Charitou et al. 2021). This creates an ongoing tension between the responsibility to protect individuals from sexual harm and the need to uphold their rights to autonomy, self‐determination, intimacy and sexual citizenship.

Research suggests that many adults with intellectual disabilities express a wish for greater support (McCarthy 2014; McGuire and Bayley 2011; Foley and Kelly 2009), and that support staff can be seen as well‐placed to offer guidance (Bates et al. 2021) as their regular, day‐to‐day contact provides insight into individuals' communication, preferences and decision‐making while fostering trusted relationships (Bigby et al. 2009; Giesbers et al. 2019; Stancliffe et al. 2000). This places them in a unique position to support relationship development, future planning and informed consent (Bates et al. 2021; Brennan et al. 2020). Both groups, however, report difficulties engaging in dialogue around sexuality (McCarthy 2014; Rushbrooke et al. 2014). This disconnect between the needs of individuals, the expectations placed on staff and the real‐world barriers they each encounter, underscores the need to gain a greater understanding of how best to support the sexual rights and well‐being of adults with intellectual disabilities. This study seeks to address this gap by exploring the dynamics of sexuality‐related conversations and the challenges experienced by those involved. In accordance with PRISMA guidelines, this review identifies and synthesises qualitative evidence on these conversations, with a focus on key facilitators and barriers. The discussion then interprets these findings through a rights‐based lens and considers implications for practice, policy and future research.

### 1.1 Perspectives of Adults With Intellectual Disabilities

Sexuality is a fundamental human right, yet adults with intellectual disabilities have historically been marginalised in discussions of their sexual autonomy (Gill 2015; Young et al. 2012). Paternalistic assumptions that position people with intellectual disabilities as asexual or childlike have restricted access to sexuality education and relationships, reflecting what has been described as ‘sexual ableism’ (Gill 2015). Although attitudes are increasingly shifting towards recognising equal rights to sexual health, relationships and expression (Charitou et al. 2021), people with intellectual disabilities remain active participants in their own sexuality (Gill 2015).

Research exploring sexuality from the perspectives of adults with intellectual disabilities consistently demonstrates their desire for greater support in expressing their sexuality and exercising their sexual rights (Frawley and McCarthy 2022; McGuire and Bayley 2011). A systematic review of 23 studies published between 2006 and 2016 found that adults with intellectual disabilities consistently sought more comprehensive sexuality and relationship education, alongside greater recognition of their sexual rights (Brown and McCann 2018). Conversations about the importance of sexuality and consent have been evidenced to reduce the incidence of sexual violence and empower individuals to speak out when they feel uncomfortable or threatened (Svae et al. 2022). Some evidence, however, suggests there are challenges for young adults with intellectual disabilities in understanding consent, along with a lack of awareness about their rights (Hart et al. 2025). Communication about sexuality also has been reported to empower individuals to understand their bodies, feelings and boundaries, which in turn assists adults with intellectual disabilities to make informed decisions about relationships, intimacy and personal safety (Svae et al. 2022). In spite of these clearly articulated needs, there is sparse information around overcoming communication difficulties on the topic of sexuality, thereby perpetuating a cycle of challenges in discussing sensitive topics.

### 1.2 Staff Perspectives

Staff attitudes and role perceptions shape opportunities for adults with intellectual disabilities to express their sexuality (Correa et al. 2022), influencing whether staff engage proactively or reactively in sexuality‐related discussions (Correa et al. 2022; Deffew et al. 2022). Staff characteristics, including age, gender and religiosity, have been suggested to influence attitudes and support practices working alongside adults with intellectual disabilities (Saxe and Flanagan 2013), with older, less trained and more religious staff tending to hold more conservative views (Deffew et al. 2022). Staff have been identified to play important roles in developing relationships (Bates et al. 2021; Schaafsma et al. 2014), future planning to promote autonomy and a sense of security (Brennan et al. 2020; de Wit et al. 2022). This dynamic may create challenges where adults with intellectual disabilities become reliant on the support and education provided by support staff to express their sexuality and engage in relationships (Rushbrooke et al. 2014; Abbott and Burns 2007).

A study in 2022 (Frawley et al. 2022) surveying Australian sexual health professionals made recommendations with the goal of addressing equity of access to sexual health information and support for adults within intellectual disability services. One of the recommendations to come from this survey was that health staff providing support to adults with intellectual disabilities are supported and prepared to provide sexual health information and engage in conversations about sexuality (Frawley et al. 2022). Staff supporting adults with intellectual disabilities in a variety of capacities, however, report receiving limited training on the topic of sexuality and feeling unsupported in their role to engage in these conversations (Frawley et al. 2022; Leclerc and Morin 2022; Charitou et al. 2021).

A systematic review of 15 papers from 2007 to 2019 (Charitou et al. 2021) exploring how staff working alongside adults with intellectual disabilities perceived their role in relation to supporting sexuality and relationships highlighted that, from a staff perspective, translating this right in practice gives rise to challenges such as a lack of training or policy guidance within the service, uncertainty regarding the remit of their role and family factors that can give rise to complications (Charitou et al. 2021).

Understanding how adults with intellectual disabilities communicate about sexuality, particularly with staff, remains a significant gap in current research. Dyer and Das Nair's (2013) review of eight studies (2001–2011) examined UK healthcare professionals' experiences discussing sexuality with adults with disabilities and found that they did not proactively discuss sexuality issues. Although this review included research pertaining to adults with intellectual disabilities, it is important to note that it covered a broad range of disabilities and thus does not fully reflect their unique experiences.

### 1.3 Rationale for the Present Study

Examining the research there appears to be a triangulation of dissonance between; the expressed requests from adults with intellectual disabilities for support, the recommendations for staff within their uniquely placed position, and the reported feedback from both adults with intellectual disabilities and staff who identified a number of challenges to engaging in conversations about sexuality. This lack of coherence through the evidence to date prevents a fuller understanding of their experiences and needs in the context of sexuality. Examining conversations about sexuality and consent provides a direct means of understanding how rights, knowledge and support are enacted in everyday practice, beyond what is stated in policy or legislation (Kelly et al. 2009). Focusing on conversational interactions allows insight into when, how and under what conditions sexual consent is addressed, revealing both opportunities for empowerment and the relational, organisational and cultural factors that shape practice. Therefore, an impetus exists to examine the research evidence on adults with intellectual disabilities discussing sexuality with staff, and the challenges faced by both parties in generating and successfully managing these conversations.

### 1.4 Aim of the Present Study

This systematic review aimed to identify and assess existing qualitative studies addressing conversations about sexuality between health and social care staff and adults with intellectual disabilities. The following questions were explored:

How frequently are conversations about sexuality between staff and adults with intellectual disabilities reported in the literature?

What factors facilitate conversations that do take place?

What factors act as barriers to these discussions in practice?

## 2 Methodology

The current review was conducted using the guidelines of Preferred Reporting Items for Systematic Reviews and Meta‐Analysis (PRISMA; Shamseer et al. 2015). This systematic review was published to the PROSPERO database on the 27th of August 2023. The protocol was revised on October 11th, 2024 to reflect that the original aim included both family members and staff, but due to resource limitations the focus of analysis was narrowed to staff only.

### 2.1 Search Strategy

The literature search was applied to the following four electronic databases: PsycINFO, MEDLINE, CINAHL and Scopus for studies published between 1st of January 2006 and 17th November 2023 with an updated review conducted on 25th of August 2025. There were four key areas of definition within the search string: (1) adults with intellectual disabilities, (2) sexuality, (3) family members and staff, (4) methods of communication. The term family was retained within the search strategy in order to maximise coverage of papers within this field, albeit papers focusing solely on family perspectives were excluded. The search strategies were decided with the support of a senior librarian and are included in Appendix S1. No geographical restrictions were applied to the search or eligibility criteria. The included studies represented a range of geographical contexts, which are reported in Table 1.

**TABLE 1:** Summary of included studies.

Authors and year	Focus of study	Country	Participant type and number	Total participants	Methods and type of analysis
Abbott and Burns (2007)	Interviews with staff and adults with intellectual disabilities to understand more about how, or if, services were able to support LGBTQ people with intellectual disabilities.	UK	Adults with ID (20); staff (71)	91	One‐on‐one interviews; grounded theory analysis
Agaronnik et al. (2020)	Exploring perceptions of caring for persons with disability from the perspective of practising physicians.	United States	Staff (42)	42	One‐on‐one interviews and focus groups; content analysis
Bates et al. (2020)	Explore the views and practices of support staff to help people with intellectual disabilities to develop relationships.	UK	Staff (26)	26	Focus group; thematic analysis
Charpentier and Carter (2023)	Explore the experiences and perspectives on romantic relationships regarding adults with intellectual disabilities involved in an inclusive supported living programme.	United States	Adults with ID (10); staff (8); parents (3)	21	One‐on‐one interviews; inductive analysis
Chou et al. (2020)	Evaluates the outcomes of a 2‐year intervention to promote sexual and reproductive health knowledge/positive attitudes and quality of life for adults with intellectual disabilities.	Taipei	Adults with ID (23); staff (19); parents (6)	48	One‐on‐one interviews and focus groups; interpretative phenomenological analysis
Deffew et al. (2022)	Explores staff attitudes and experiences of people with an intellectual disability's expression of relationships and sexuality across service providers.	Republic of Ireland	Staff (86)	86	Survey questionnaire; thematic analysis
Grace et al. (2020)	Explore service users' accounts of the messages around sexuality conveyed by staff within forensic intellectual disability services.	UK	Adults with ID (8)	8	One‐on‐one interviews; critical discourse analysis
Holler and Bondorevsky‐Heyman (2023)	Exploring the dynamics of intimate relations for adults with intellectual disabilities from the perspective of social workers employed in residential settings.	Israel	Staff (15)	15	One‐on‐one interviews; thematic analysis
Kahonde and Johns (2022)	Explore and describe the knowledge and awareness of risk to sexual violence among adults with intellectual disabilities and to understand their perceptions and experiences of risk.	South Africa	Staff (27)	27	Focus group; thematic analysis
Lafferty et al. (2012)	Examine the attitudes of family carers, direct care staff and trained professionals (such as social workers and nurses) to the provision of Relationship Sexuality Education (RSE) for teenagers and adults with intellectual disabilities	Northern Ireland	Staff (48) and family caregivers (48)	96	One‐on‐one interviews and focus groups; content analysis
Lam et al. (2022a)	Explores the experiences of people with intellectual disabilities on their need for an intimate or romantic relationship and sexual expression, along with the sociocultural factors that potentially influence these experiences.	Hong Kong	Adults with ID (5)	5	One‐on‐one interviews; interpretative phenomenological analysis
Lam et al. (2022b)	Explore their experiences of and attitudes towards the sexual needs of people with intellectual disabilities from the perspective of staff	Hong Kong	Staff (7)	7	One‐on‐one interviews; interpretative phenomenological analysis
Maguire et al. (2019)	This research aimed to explore support workers understanding of their role supporting the sexuality of people with learning disabilities.	UK	Staff (6)	6	One‐on‐one interviews; interpretative phenomenological analysis
McCarthy et al. (2022)	This paper sought to explore with adults with intellectual disabilities what kinds of support they wanted in their desire to form loving relationships.	UK	Adults with ID (40)	40	One‐on‐one interviews; thematic analysis
Neuman (2020)	This research explored direct support staff attitudes and role perception in the area of couple relationships between adults with intellectual disabilities	Israel	Staff (40)	40	One‐on‐one interviews; thematic analysis
Pariseau‐Legault et al. (2019)	This study aimed to explore the ethical implications of support workers' experiences concerning sexuality in the context of intellectual disabilities.	Canada	Staff (6)	6	One‐on‐one interviews; interpretative phenomenological analysis
Rojas et al. (2016)	As a piece within a broader study, this research aimed to explore the ideas for adults with intellectual disability about sexuality and their sexual lives.	Spain	Adults with ID (16)	16	One‐on‐one interviews; thematic coding
Santinele Martino (2022)	This paper aimed to demonstrate how religion can shape the intimate lives of people with intellectual disabilities and the practices of direct care workers providing care to adults with intellectual disabilities.	Canada	Staff (6)	6	One‐on‐one interviews; thematic analysis
Schaafsma et al. (2017)	This study was conducted to assess the perspectives of people with intellectual disabilities on sexuality‐related topics.	The Netherlands	Adults with ID (20)	20	One‐on‐one interviews; thematic coding
Stoffelen et al. (2018)	Aim is to gain insight into the lives of a specific cohort of women with ID who have sexual feelings for other women, or who identify themselves as lesbian or bisexual, and are living in the Netherlands.	The Netherlands	Adults with ID (10)	10	One‐on‐one interviews; thematic coding
Thompson et al. (2014)	This paper explores the perspectives of disability service managers and clinicians about sexual health and service provision for adults with intellectual disabilities.	Australia	Staff (31)	31	One‐on‐one interviews; grounded theory analysis
Turner and Crane (2016)	To describe the lived sexual experience of five adults with intellectual disabilities by exploring the meaning they attach to their sexual lives, and by examining their social‐sexual voice.	United States	Adults with ID (5)	5	Two interviews and home observation; discourse analysis
Wickström et al. (2020)	Gain a deeper understanding of staff's experiences and perceptions regarding sexual and reproductive health and rights (SRHR) for young people with intellectual disabilities.	Sweden	Staff (20)	20	Focus group; content analysis

Abbreviations: ID = intellectual disabilities; LGBTQ = lesbian, gay, bisexual, transexual, queer; RSE = relationship sexuality education; SRHR = sexual and reproductive health and rights.

### 2.2 Inclusion and Exclusion Criteria

To investigate and identify the context surrounding conversations about sexuality, it was decided to include only qualitative study designs in this review. Mixed‐method design studies were included during screening; however, only qualitative data were extracted for the purpose of this review.

The rationale for investigating qualitative data was firstly that qualitative studies can provide a deeper understanding of participants' interactions, behaviour and experiences, and secondly to permit the identification of data around this complex area in greater depth across a variety of contexts (Bryman 2015; Lachal et al. 2018). There is some disagreement around integrating individual qualitative studies (Dixon‐Woods et al. 2001); however, this current research design reflects the belief that qualitative research can be synthesised to build a greater understanding of phenomena to inform both policy and practice (Thomas and Harden 2008). Inclusion criteria were defined as follows:

– Any range of communication (e.g., verbal, Augmentative and Alternative Communication, sign language, visual, written supports etc.), reporting the presence of staff discussing sexuality with adults with intellectual disabilities and their experience of doing so including discourse, safeguarding responses, education, relationships and sexuality education (RSE), sexual health promotion.
– Participants included health and social care professionals (e.g., direct support workers, clinicians, educators, crisis support staff) who were supporting adults with intellectual disabilities in a professional context.
– Studies where participants were adults with intellectual disabilities were also included, where these participants referred to their interactions with staff.
– Primary research studies which are peer‐reviewed research published in journals.
– Studies published from 2006 onwards, as the definition of sexuality used in this review was established by the World Health Organisation in 2006. The WHO conceptualisation of sexuality was used to inform eligibility, with studies considered eligible where sexuality‐related content extended beyond sexual health and included domains such as relationships, intimacy, sexual expression, identity or reproduction.
– Qualitative and mixed methods studies.

Articles were excluded where:

– Studies that involved or referenced individuals with intellectual disabilities under the age of 18 were excluded, as the aim of this review was to explore the experiences of adults. In cases where research included both minors and adults, the study was excluded if data specific to participants aged 18 and over could not be separately identified and extracted.
– Peer‐reviewed systematic reviews and editorials, presentations and non‐peer‐reviewed articles.

Non‐English language studies and grey literature (such as books, dissertations, conference abstracts).

– Reports of sexual abuse pertaining solely to safeguarding, decision‐making and legal intervention.

Additionally, hand‐searching reference lists of the included papers was applied to further identify articles which met the inclusion criteria.

### 2.3 Quality Assessment and Researcher Reflexivity

Quality assessment involved restricting inclusion to peer‐reviewed journal articles. Critical appraisal was subsequently conducted by the first author using the Critical Appraisal Skills Programme for Qualitative Research (CASP 2024). Each study was evaluated across 10 questions, rated as Low, High or Unclear risk. Consistent with CASP guidance, the tool was not used as a scoring system and no studies were excluded on this basis; rather, appraisal served to identify methodological limitations across the included articles.

Reflexivity is essential in qualitative research to mitigate the influence of researcher bias on findings (Popay et al. 2006). The lead author's prior clinical experience with staff and adults with intellectual disabilities was addressed through reflective journaling throughout analysis and independent data extraction by a second reviewer across 25% of studies, with comparison against the lead author's extraction to establish inter‐rater reliability.

## 3 Results

### 3.1 Study Selection

Following the PRISMA flow guidelines (as seen in Figure 1), there were four distinct steps to the selection procedure: (1) identification, (2) screening, (3) eligibility and (4) extraction.

**FIGURE 1:**
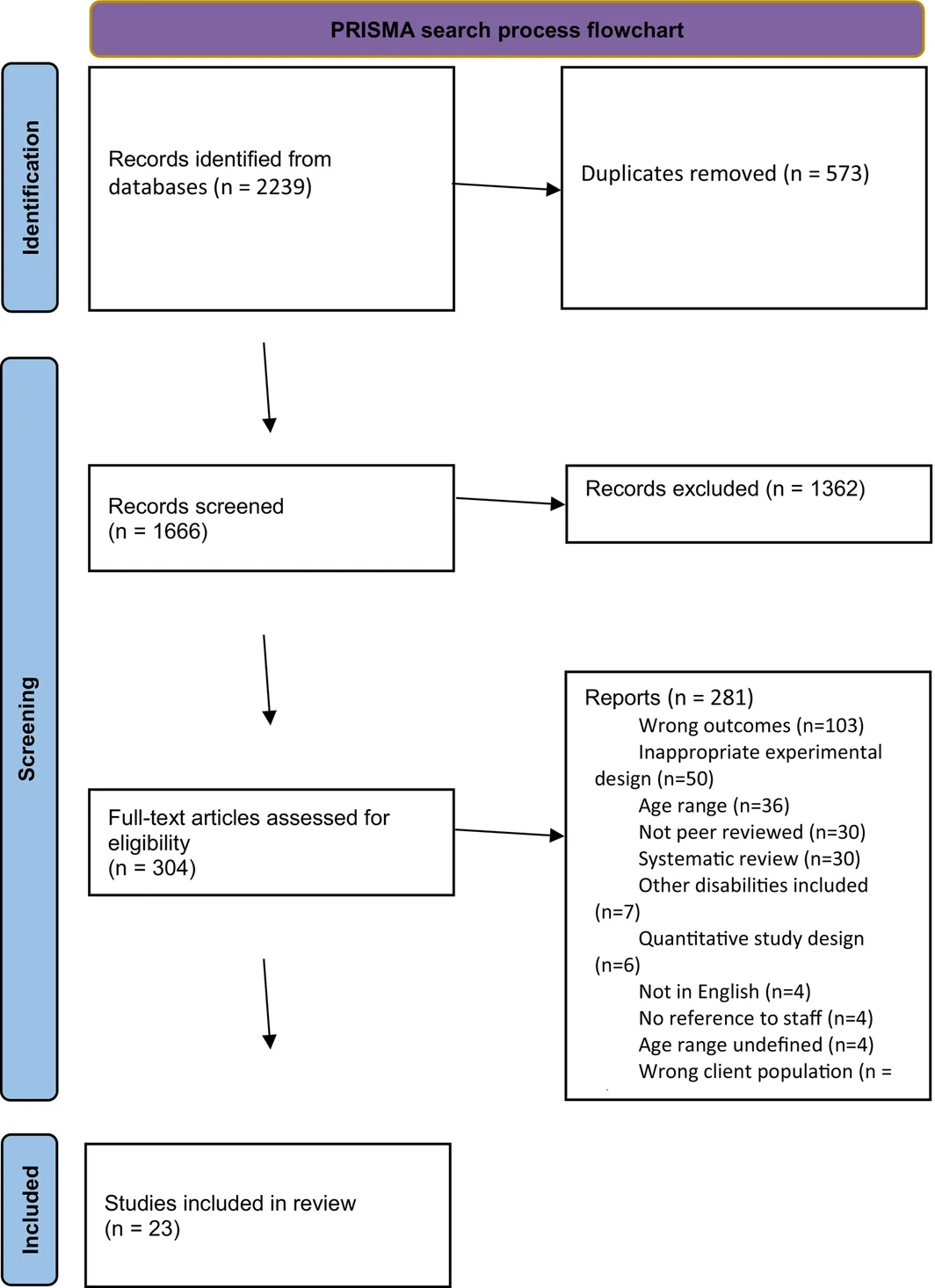
PRISMA flowchart.

The identification phase retrieved articles through the search string which yielded 2239 articles. Duplicates (573) were removed which resulted in 1666 articles to be screened for the next stage of the selection process. During the screening phase, first and second authors each independently screened all titles and abstracts in line with inclusion and exclusion criteria using the software Covidence. The agreement ratio was 87%, with disagreements discussed until a consensus was established. Eligibility stage involved full‐text review of 304 articles by inspecting their methodologies and research, with the second reviewer completing 25% of the full text screening and an agreement rate of 88%.

The process identified 26 of these articles as meeting the inclusion criteria. Three initially eligible articles were then excluded due to duplicated data found in other included studies. In such cases, preference was given to the article presenting the most comprehensive dataset relevant to the research question. On the 25th of August 2025, prior to submission of this thesis, the search string was reapplied to review most recent articles, if any, published since the initial search on 17th November 2023. This yielded 47 additional articles, all of which were excluded at the screening phase. Consequently, 23 articles were eligible for inclusion in the present review.

The quality appraisal of included studies using the CASP tool indicated that the highest proportion of studies (*n* = 9) were of high quality, with a smaller number classified as medium quality (*n* = 8) and the lowest amount of studies falling into the low‐quality category (*n* = 6). This suggests that of the studies included in the review, 26% are of low quality. The key issues identified through this appraisal process were primarily related to reporting rather than fundamental flaws in study design. Further details of the ratings are outlined in Table 2.

**TABLE 2:** Quality ratings table.

Study ID	1. Aim	2. Methodology	3. Design	4. Recruitment	5. Data collection	6. Researcher role	7. Ethics	8. Data analysis	9. Findings	10. Impact	Total score	Global rating
Abbott 2007	0.5	1	1	1	1	0	0	1	1	1	7.5	Medium
Agaronnik 2020	0.5	0.5	0.5	1	1	0.5	0	0.5	1	1	6.5	Low
Bates 2020	1	1	0.5	1	1	0.5	1	1	1	1	9	High
Charpentier 2023	1	1	0.5	1	1	1	1	1	1	1	9.5	High
Chou 2020	1	1	1	0.5	0.5	0	0.5	0.5	0.5	1	6.5	Low
Deffew 2022	1	1	1	1	0.5	0	0.5	1	1	1	8	Medium
Grace 2020	1	1	1	1	1	1	1	1	1	1	10	High
Holler 2023	1	0.5	0	1	0.5	0	1	0.5	0	1	5.5	Low
Kahonde 2022	1	1	1	1	1	0.5	1	1	1	1	9.5	High
Lafferty 2012	1	1	1	1	1	1	1	1	1	1	10	High
Lam 2022a	1	1	1	1	1	0	1	0.5	0.5	1	8	Medium
Lam 2022b	0.5	1	1	1	1	0	1	0.5	1	1	8	Medium
Maguire 2019	1	1	1	1	1	0	1	1	1	1	9	High
McCarthy 2022	1	1	1	1	1	1	1	1	1	1	10	High
Neuman 2020	1	1	1	1	1	0	1	1	1	1	9	High
Pariseau‐Legault 2019	1	1	1	1	1	0.5	1	1	1	0.5	9	High
Rojas 2016	0.5	1	0.5	1	0.5	0.5	1	1	0.5	0.5	7	Low
Santinele Martino 2022	1	1	1	0.5	0.5	0	0.5	0.5	0.5	0.5	6	Low
Schaafsma 2017	1	1	1	1	1	0	1	0.5	1	1	8.5	Medium
Stoffelen 2018	1	1	0	1	0.5	0	1	0.5	1	1	7	Low
Thompson 2014	1	1	1	1	1	0.5	1	0.5	0.5	1	8.5	Medium
Turner 2016	0.5	1	1	0.5	1	1	0.5	1	1	0.5	8	Medium
Wickstrom 2020	1	1	0	1	1	0	1	1	1	1	8	Medium

*Note:* Scoring system: yes—1 point, unsure—0.5 points, no—0 points; high quality: scores 9–10, medium quality: scores 7.5–9, low quality: < 7.5.

### 3.2 Characteristics of Included Studies

All studies bar one used qualitative methodology; one study used a mixed methods design (Deffew et al. 2022). Fourteen studies using one‐to‐one interviews; three employed focus groups; two used both group interviews and one‐to‐one interviews; one included a questionnaire with open ended questions that was analysed qualitatively; one included a repeated interview and a home observation; one included an interview post intervention; and one study conducted one‐to‐one interviews including the use of vignettes.

A variety of methods were used for analysis, including thematic analysis (9), interpretative phenomenological analysis (5), grounded theory (3), discourse analysis (2), three‐step coding analysis (2), thematic coding (1) and content analysis (1).

Populations within the articles included staff (11) and adults with intellectual disabilities (9). One article included a mixed participant group of both adults with intellectual disabilities and staff (1); another included adults with intellectual disabilities, families and staff (1); and finally, one included staff and families (1). A total of 672 participants were included in the papers included in this review, with 458 (68%) participants being staff and 157 (23%) participants adults with intellectual disabilities. The remaining 57 (9%) participants were family and caregivers; however, as noted in the inclusion criteria, only data from the perspectives of adults with intellectual disabilities and staff were included within this review.

Studies were carried out across a number of geographical regions, including United Kingdom (5), United States (3), Canada (2), Hong Kong (2), Israel (2), the Netherlands (2), Australia (1), Northern Ireland (1), Republic of Ireland (1), South Africa (1), Spain (1), Sweden (1) and Taipei (1). General study information can be found in Table 3.

**TABLE 3:** Conversation occurrence.

Real conversation—presence	Real conversation—absence	Hypothetical	Total
39	3	33	75

### 3.3 Results of Narrative Synthesis

Data were analysed using a narrative synthesis approach (Popay et al. 2006). Data containing original quotes within the text were extracted, with details of the conversation reviewed. To address Research Question 1, the context of conversations was first established. Two categories were applied:

- Real conversations are defined as conversations about sexuality that were explicitly documented within an article and further subdivided into two types:
  1. The ‘presence’ of a conversation denotes cases where staff referred to situations where an actual conversation about sexuality occurred with adults with intellectual disability.
  2. The ‘absence’ of a conversation denotes cases where staff referred to situations where a conversation about sexuality with adults with intellectual disability was deliberately avoided.
- Hypothetical conversations are defined as conversations about sexuality that were described within an article as imagined or having the potential to occur but were not verified within the article as having occurred.

To address Research Questions 2 and 3, relevant text was extracted, coded and tabulated using narrative synthesis (Popay et al. 2006), with data subsequently grouped into meaningful themes (Thomas et al. 2012). To enhance reliability, a second rater independently coded data from 26% of included articles (*n* = 6), yielding an inter‐rater agreement of 87%, indicating strong coding consistency. Discrepancies were resolved through discussion until consensus was reached. Following data extraction, conversations were further categorised as proactive or reactive to contextualise the timing of interactions.

#### 3.3.1 Prevalence of Conversation Occurrence

Across 23 articles, a total of 75 real conversations were referenced. The articles included in this review span a period of approximately 16 years, from Abbott and Burns (2007) to Charpentier and Carter (2023) and Holler and Bondorevsky‐Heyman (2023). While earlier studies are less frequent, there is a noticeable increase in publications from 2019 onwards with multiple studies published each year (e.g., Bates et al. 2020; Grace et al. 2020; Lam et al. 2022a, 2022b; McCarthy et al. 2022).

Of these, 39 conversations were described as conversations having taken place (‘presence’) with a further three being explicitly described where the conversation was avoided (‘absence’). Together, these figures were combined and classified as ‘real’ conversations (*n* = 42). A further 33 conversations were reported as being classified as hypothetical.

Real conversations that had actually taken place (*n* = 39) were further categorised by approach: proactive conversations (*n* = 24) and reactive conversations (*n* = 15). Proactive conversations were undertaken prior to sexual expression, for example:

He's warned me about things that could happen. (Grace et al. 2020)

Reactive conversations were identified as having taken place following an expression of sexuality such as:

If there's a problem related to sexuality, it will be addressed. If there is no problem, it will not be discussed and we will work on something else instead. (Pariseau‐Legault et al. 2019)

Absence of real conversations (*n* = 3) referenced incidences where a conversation about sexuality was intentionally avoided:

in a sense we did palm it off, but there's nothing in our policies as, it comes back to policies and procedures, to say what we can and can't do or whether we cross the line. (Thompson et al. 2014)

Hypothetical conversations (*n* = 33) involved imagined or potential scenarios, with the specificity of a real‐world context remaining ambiguous:

I wouldn't push them into anything, but I also wouldn't necessarily shy from anything (Charpentier and Carter 2023).

Within the subset of presence conversations, proactive discussions (initiated prior to sexual expression) accounted for 24 instances, whereas reactive conversations (occurring in response to an expression of sexuality) accounted for 15 instances. These findings suggest that opportunities for sexuality‐related dialogue are limited, with interactions tending to occur reactively rather than as part of routine, proactive support.

While these figures provide an overview of the frequency of dialogue, greater insight may be gained from exploring the quality, content and themes that characterise these interactions. Conversations were then examined to identify factors mentioned in the studies that facilitated or hindered these discussions.

#### 3.3.2 Facilitators of Conversations

Five themes for facilitators of conversation were identified: advocating, risks and safety, organisational structure, staff confidence and positive relationships.

Self‐advocacy by adults with intellectual disabilities was identified as a facilitator across 11 articles (Abbott and Burns 2007; Charpentier and Carter 2023; Grace et al. 2020; Holler and Bondorevsky‐Heyman 2023; Kahonde and Johns 2022; Lam et al. 2022a; Maguire et al. 2019; McCarthy et al. 2022; Pariseau‐Legault et al. 2019; Thompson et al. 2014; Turner and Crane 2016), encompassing curiosity‐driven enquiry, goal‐directed communication, and seeking clarification on rules. This is shown to manifest in practice when adults proactively ask staff questions to understand their own bodies, feelings, and boundaries, explicitly communicate their desires and needs to form intimate, loving or romantic relationships, and engage with staff to navigate institutional regulations or warnings regarding physical contact. Three papers explicitly noted a perceived onus on adults with intellectual disabilities to initiate conversations about sexuality instead of staff (Thompson et al. 2014; Maguire et al. 2019; Abbott and Burns 2007). Risk and safety concerns prompted conversations in eight articles (Bates et al. 2020; Charpentier and Carter 2023; Deffew et al. 2022; Grace et al. 2020; Lam et al. 2022b; Maguire et al. 2019; Pariseau‐Legault et al. 2019; Thompson et al. 2014), particularly in response to behaviours perceived as inappropriate, with formal procedures further facilitating dialogue when concerns arose retrospectively. Organisational structure was noted as a conversation facilitator in seven articles (Abbott and Burns 2007; Agaronnik et al. 2020; Bates et al. 2020; Charpentier and Carter 2023; Chou et al. 2020; Grace et al. 2020; McCarthy et al. 2022). Clearly defined organisational rules, privacy rights and targeted education (such as Relationship and Sexual Education [RSE] training and specialist sexuality services) created structured opportunities for dialogue and fostered a sense of normalcy around the topic. Staff confidence, noted in four articles (Charpentier and Carter 2023; Grace et al. 2020; Lam et al. 2022b; Wickström et al. 2020), was enhanced by clear procedural frameworks such as safeguarding policies, supporting both proactive and responsive conversations. Where *positive relationships* were established between staff and adults with intellectual disabilities, this facilitated conversations in three articles (Charpentier and Carter 2023; Kahonde and Johns 2022; McCarthy et al. 2022), which fostered openness to discussing sexuality, with gender dynamics also noted as influencing conversational comfort.

#### 3.3.3 Barriers to Conversations

Six themes for facilitators of conversation were identified: fear and avoidance, organisational structure and resourcing, role tension, lack of relational safety, taboo subject and parental influence.

Fear and avoidance were identified as barriers to conversation across 14 articles, affecting both staff and adults with intellectual disabilities (Abbott and Burns 2007; Charpentier and Carter 2023; Deffew et al. 2022; Grace et al. 2020; Holler and Bondorevsky‐Heyman 2023; Lafferty et al. 2012; Lam et al. 2022a; Lam et al. 2022b; Maguire et al. 2019; Pariseau‐Legault et al. 2019; Rojas et al. 2016; Stoffelen et al. 2018; Thompson et al. 2014; Wickström et al. 2020). Concerns included working outside service policies, fear of colleague backlash and punitive consequences. Adults with intellectual disabilities additionally reported avoiding conversations for fear of losing their accommodation, with safeguarding processes identified as a specific deterrent. The perception by individuals of the impact of organisational structure and resourcing was perceived as a barrier in 11 articles (Abbott and Burns 2007; Charpentier and Carter 2023; Deffew et al. 2022; Grace et al. 2020; Holler and Bondorevsky‐Heyman 2023; Lafferty et al. 2012; Pariseau‐Legault et al. 2019; Santinele Martino 2022; Thompson et al. 2014; Turner and Crane 2016; Wickström et al. 2020), reflecting two conflicting approaches: overly restrictive rules or an absence of formal procedures. Staff reported feeling constrained by workplace cultures, which could be shaped by managerial precedent, in addition to resourcing pressures frequently resulting in adults with intellectual disabilities being expected to initiate discussions.

We're busy doing a million other things, and there's no time for it. (Holler and Bondorevsky‐Heyman 2023)

Role tension was identified in nine articles (Abbott and Burns 2007; Charpentier and Carter 2023; Deffew et al. 2022; Grace et al. 2020; Holler and Bondorevsky‐Heyman 2023; Lam et al. 2022b; Maguire et al. 2019; Pariseau‐Legault et al. 2019; Wickström et al. 2020), with staff reporting persistent uncertainty regarding the boundaries of their role, who should initiate conversations, and their own knowledge and competence.

I don't know what I can't open up or not, you know. And I also don't know what kind of leverage or responsibility would be assigned back to me when and if I open up these conversations, right. (Charpentier and Carter 2023)

Embarrassment and discomfort, particularly around specific topics such as masturbation, were also reported. Lack of relational safety was observed to act as a barrier for adults with intellectual disabilities to conversations in seven articles. (Abbott and Burns 2007; Grace et al. 2020; Lam et al. 2022a; McCarthy et al. 2022; Stoffelen et al. 2018; Thompson et al. 2014; Turner and Crane 2016). Adults with intellectual disabilities reported a distrust of certain staff, perceptions of aversive attitudes towards their sexuality and a sense that staff lacked genuine interest, collectively discouraging initiation of conversation. The perception of sexuality as a taboo subject was identified as a barrier to conversation in four studies (Abbott and Burns 2007; Charpentier and Carter 2023; Lam et al. 2022a; Wickström et al. 2020) with both groups experiencing shame and embarrassment around the subject. The theme of parental influence was identified in three articles (Agaronnik et al. 2020; Lam et al. 2022b; Turner and Crane 2016) represented a further barrier, with staff deferring to parental authority over adults' expressed autonomy and reporting that navigating parental involvement created additional complexity and reluctance to engage.

## 4 Discussion

This review set out to explore conversations about sexuality between adults with intellectual disabilities and staff. Findings of the current study indicate a complex interplay of individual, organisational and societal factors shaping these interactions.

### 4.1 Prevalence of Conversation Occurrence

Across the 23 qualitative studies reviewed, 75 documented occurrences of sexuality‐related dialogue were identified within the qualitative evidence base, representing 39 confirmed real‐world interactions. The distribution of the included studies highlights both trends and gaps in the literature. The articles included in this review span a period of approximately 16 years, from Abbott and Burns (2007) to Charpentier and Carter (2023) and Holler and Bondorevsky‐Heyman (2023). The figures equate to an average of approximately two conversations between staff and service users regarding sexuality per year appearing in the literature. While research on sexuality conversations involving adults with intellectual disabilities has been published intermittently since 2007, there is a clear clustering of studies from 2019 onwards, suggesting increasing scholarly attention to this topic in recent years (e.g., Bates et al. 2020; Grace et al. 2020; Lam et al. 2022a, 2022b; McCarthy et al. 2022; Charpentier and Carter 2023). While this suggests growing attention to the topic, the overall volume of research remains relatively limited. The concentration of more recent studies may reflect changing policy contexts locally, evolving societal attitudes towards sexuality and disability (Correa et al. 2022) and heightened awareness of staff training needs (de Wit et al. 2022). The uneven distribution, however, indicates that evidence is beginning to emerge but still insufficiently robust to draw comprehensive, longitudinal, or internationally generalizable conclusions.

The presence of 33 hypothetical conversations indicates ongoing uncertainty or discomfort with addressing sexuality directly. This finding reflects previous evidence indicating that professionals frequently report a lack of confidence and training to engage in real sexuality discussions, leading to theoretical rather than practical responses (Pérez‐Curiel et al. 2024). This review's identification of communication challenges reinforces recent calls for comprehensive, rights‐based approaches, particularly given that ‘adults with intellectual disabilities need education and support to express their sexuality and to meet individual needs’ (Brown and McCann 2018, 124).

### 4.2 Facilitators

Facilitators were diverse but consistently pointed to the importance of relational, organisational and individual factors. At an individual level, the capacity of adults with intellectual disabilities to advocate for themselves emerged as a consistent facilitator of conversations about sexuality. Evidence indicates that when individuals proactively seek information, clarification or support regarding sexual matters, staff are more likely to engage in meaningful dialogue. Azzopardi‐Lane and Callus (2014) proposed conversations about sexuality as a method of advocating for oneself. As demonstrated in this review, when adults with intellectual disabilities engaged in self‐advocacy, expressed curiosity or articulated specific goals, this further facilitated meaningful engagement. The United Nations Convention on the Rights of Persons with Disabilities (UNCRPD) upholds the rights of adults with intellectual disabilities to express their sexuality through Articles 23 and 25 by promoting autonomy and access to inclusive sexual education and support (United Nations 2006). Providing opportunities for adults with intellectual disabilities to ask questions about sexuality and providing clarity around boundaries is identified as an essential and ongoing task within services.

Staff occupy a pivotal position in the lives of adults with intellectual disabilities, with their practices either facilitating or constraining sexual expression (de Wit et al. 2022; Charitou et al. 2021). Through their influence over privacy, social opportunities and community participation, staff can shape access to the conditions necessary for intimacy (Schaafsma et al. 2014; Perez‐Curiel et al. 2023). However, a duty‐of‐care emphasis on safeguarding and risk management may prioritise protection over autonomy (Pelleboer‐Gunnink et al. 2021; Jingree 2014), potentially making sexual rights contingent on staff attitudes and organisational risk aversion rather than recognising them as inherent rights.

At an organisational level, practices that respected privacy, allocated time and resources, and formalised opportunities for discussion enabled staff to feel legitimised in addressing sexuality. The theme of risks and safety, reflecting the use of a lens of risk or behavioural concern, is supported by Deffew et al.'s (2022) findings of staff interactions around sexuality limited to the context of curtailing ‘inappropriate behaviours’ through an individual's ‘Behaviour Support Plans’. This finding aligns with previous literature indicating that staff uncertainty and anxiety can inhibit proactive engagement with sexual health topics, particularly in the absence of structured guidance or training (de Wit et al. 2022; Pérez‐Curiel et al. 2024). Although the rights of adults with intellectual disabilities are increasingly acknowledged, frustration, uncertainty, and stigma persist (Pérez‐Curiel et al. 2024). Current evidence reveals that support staff often lack training to address sexual health questions, leading individuals with intellectual disabilities to rely on subjective assessments of staff competence rather than formal qualifications when seeking sexual health support (de Wit et al. 2022). Staff training and targeted education were found to normalise sexuality as a topic and increased confidence, though such efforts were often ad hoc or reliant on external specialists. This finding is consistent with contemporary studies revealing that sexuality education for support staff remains fragmented and inconsistent across care settings, with many organisations lacking systematic training programmes (Gil‐Llario et al. 2023; Pérez‐Curiel et al. 2024). This reliance on external specialists may inadvertently reinforce the idea that conversations about sexuality fall outside staff's remit, pathologising sexuality within a medicalisation framework and perpetuating barriers to routine discussion (de Wit et al. 2022). When sexuality discussions are relegated to specialist domains, this may also reinforce organisational cultures that view sexuality as problematic (de Wit et al. 2024), rather than approaching from a rights‐based perspective positioning sexuality as a fundamental aspect of human experience (Whittle and Butler 2018).

Finally, the quality of the relationship between staff and adults with intellectual disabilities was identified as a pivotal facilitator. Positive, trusting relationships can foster safe and respectful dialogue about sexuality (Charpentier and Carter 2023; Kahonde and Johns 2022; McCarthy et al. 2022), with staff reporting greater comfort when rapport is established and gender concordance further supporting engagement in sensitive discussions (McCarthy et al. 2022). However, what constitutes a ‘positive’ relationship, and for whom, warrants critical consideration. Given adults' reliance on staff for personal care, daily activities and community participation, trust may not be inherently reciprocal and can coexist with significant relational power imbalances (Bourke et al. 2024). Thus, while trust and rapport are important to person‐centred care and sexual rights, they may also enable staff to retain disproportionate influence over decisions regarding sexuality and intimacy (de Wit et al. 2022; Whittle and Butler 2018).

### 4.3 Barriers

A range of persistent and deeply embedded barriers were evident across the literature. Fear—experienced by both staff and adults with intellectual disabilities—emerged as a deterrent to conversations. Sexuality was associated with punitive consequences such as threats to housing or relationships, thereby shaping an environment where reactive interventions were more common than proactive engagement. Similar to the existing research, staff uncertainty was a pervasive theme particularly with regard to their role; the boundaries of professional responsibility, and the appropriate way to engage in conversations about sexuality (Greenwood and Wilkinson 2013; Devik et al. 2025). This was compounded by a widespread lack of training, discomfort with the topic, and fears of professional repercussions such as potential backlash from colleagues or management, which echoes previous findings (e.g., de Wit et al. 2022; Brown and McCann 2018).

Organisational structures created obstacles as some service cultures were shaped by risk‐averse/paternalistic policies, with a tension observed echoing the growing openness towards the topic of sexuality versus the lack of policies and guidance for implementation (Perez‐Curiel et al. 2023). Findings indicated that staff often deferred to parental preferences over those of adults with intellectual disabilities, reflecting the broader influence of the mesosystem—defined by Bronfenbrenner (1979) as the interconnections between different microsystems, such as family, service providers, and community networks. For example, research has shown that while service leaders emphasise the importance of family involvement, few initiatives have been developed to engage families directly (Boland and Guerin 2022). Such patterns illustrate broader challenges around how services prioritise competing perspectives and how staff negotiate the balance between family influence and individual autonomy.

### 4.4 Implications for Staff and Services

The findings of this review highlight the need for development within service‐based policy in how conversations about sexuality are embedded within disability support services, which, based on the present findings, can often be reactive or risk averse. Policies should clearly define the roles and responsibilities of staff in facilitating these discussions and be supported by organisational leadership that promotes a culture of openness and inclusion. Effective service and policy development should actively engage adults with intellectual disabilities, ensuring that their perspectives and lived experiences substantively inform the design and implementation of supportive environments (Belperio et al. 2024). A recent scoping review by McGrath et al. (2025) identified key threats to sexual autonomy for adults with intellectual disabilities, including assumptions of vulnerability, sexual incompetence, and normative expectations of heteronormativity and asexuality. Establishing clear and inclusive policies can help address these barriers by providing staff with defined guidance while foregrounding the rights of adults with intellectual disabilities. Such measures also support self‐advocacy and contribute to the creation of more empowering and equitable service contexts.

As has been evidenced in previous reviews, the present review reinforces that further education on sexuality is imperative (Charitou et al. 2021; Chrastina and Večeřová 2020; Brown and McCann 2018). Suggestions have been made to adapt education around sexuality for staff, so that it is ongoing, facilitates reflective practice and is modelled by senior staff members (Charitou et al. 2021), thereby increasing the knowledge base and confidence for staff, in addition to addressing emotional preparedness, values, and role clarity. Integrating this training as a regular component of staff roles, rather than positioning it solely as a specialist topic, may help to promote greater consistency and create more respectful opportunities for discussion (van Oorsouw et al. 2009).

Additionally, this research has identified the significant role parents continue to play within the lives of adults with intellectual disabilities. Providing parents with resources and materials has been suggested as an aid to discuss these issues with their relatives (Lafferty et al. 2012), with recommendations emphasising the importance of centring the adult in decision‐making, rather than defaulting to family control (Sullivan and Heng 2018).

### 4.5 Strengths and Limitations

This review's adherence to PRISMA guidelines enhances transparency and rigour in study identification and synthesis. It represents one of the first systematic attempts to map evidence on sexuality‐related conversations between staff and adults with intellectual disabilities. Notably, only 39% of included studies were rated high quality, underscoring the urgent need for more rigorous research in this area. Quality appraisal was conducted systematically using established frameworks (Popay et al. 2006), supporting consistency and credibility, though the absence of a second rater in this process represents a limitation.

Several limitations warrant acknowledgement. Limitations of the included studies included inconsistent reporting of participant age, which complicated eligibility decisions, and limited consideration of gender, age, sexual orientation and cultural background, thereby potentially overlooking diversity in experiences among adults with intellectual disabilities. The evidence base was also disproportionately drawn from high‐income, Westernised contexts, limiting cross‐cultural generalisability. A second rater was not included in the CASP quality assessments, which is a limitation; however, the review followed best‐practice guidance for synthesising qualitative and non‐randomised evidence (Popay et al. 2006). Limitations arising from the present review included a lack of differentiation between levels of intellectual disability, which may have influenced findings given variability in cognitive and communication abilities. Additionally, focus on English‐language, peer‐reviewed qualitative and mixed‐methods studies may have further narrowed the breadth of evidence captured.

### 4.6 Research Recommendations

Based on the findings of this review, research co‐designed with adults with intellectual disabilities is especially warranted to ensure that their perspectives directly inform service frameworks and decision‐making processes (Strnadová and Walmsley 2018). The findings of this review reflect consistent calls for enhanced staff education (Brown and McCann 2018; de Wit et al. 2022). Future studies may benefit from exploring the impact of reflective training interventions for staff that include mentoring, peer learning and values‐based reflection. Future research should directly involve adults with intellectual disabilities in exploring how they experience, understand, and engage in conversations about sexuality across various settings (e.g., supported living, day services, family homes), including exploration of how accessible communication strategies can be implemented for to develop tailored communication materials (van der Meer et al. 2017). Studies should investigate what makes these conversations feel safe, empowering or uncomfortable, using participatory or inclusive research methods (de Wit et al. 2024). Further investigation should examine how organisational norms and cultures are perceived by adults with intellectual disabilities, particularly how these influence their willingness or ability to discuss sexuality. Future research should explore how service environments either support or silence sexual expression, from the service user's perspective.

From a service perspective, further research is needed to explore how service‐level policies can more proactively support conversations about sexuality in ways that are rights‐based rather than reactive or risk‐driven. Studies should examine the development and implementation of policies that clearly define staff roles, boundaries, and responsibilities, with particular attention to the influence of organisational culture and leadership in shaping staff practice (Hatton et al. 1999). It may be helpful to frame the research around the inclusion of relationships and sexuality within person‐centred planning goals, how it is implemented and discussed and the outcomes for adults with intellectual disabilities and staff (Brown‐Hall 2018). Finally, given the continued centrality of parental influence, further work should explore how services can equip and engage families in supporting open and inclusive discussions about sexuality, including the development and testing of tailored resources and interventions (Bates et al. 2021; de Wit et al. 2022; Donnelly et al. 2023).

## 5 Conclusion

This systematic review and narrative synthesis explored the occurrence and context of sexuality‐related conversations between staff and adults with intellectual disabilities. Facilitators included supportive organisational structures, clear procedural guidance, trusting relationships and self‐advocacy by adults with intellectual disabilities. Barriers were pervasive, encompassing fear, stigma, role ambiguity and risk‐averse service cultures, with staff frequently prioritising protective and parental perspectives over individuals' expressed needs. This reflects an enduring tension between the recognition of sexual rights and risk‐driven models of support. Findings underscore the need for sustained staff training, greater role clarity and the development of rights‐based policies to facilitate open, empowering dialogue and address systemic discomfort surrounding sexuality in service provision.

## Funding

The authors have nothing to report.

## Conflicts of Interest

The authors declare no conflicts of interest.

## Supporting information


**Appendix S1:** SR search string.

## Data Availability

The data that support the findings of this study are available in the Supporting Information of this article.
