# Supplementary material for: Exploring How Staff Engage in Conversations Around Sexuality With Adults With Intellectual Disabilities: A Qualitative Systematic Review
Source: J Appl Res Intellect Disabil. 2026 Oct 20;39(5):e70319. doi: 10.1111/jar.70319 (PMC13681242; doi:10.1111/jar.70319)
Supplement: Supplementary file 1 [file JAR-39-e70319-s001.docx]

**Psycinfo:**

(( Intellectual Disabilit* OR "Intellectual Development Disorder" OR "Down's Syndrome" OR “Downs Syndrome” OR “Developmental Disabilit*” OR “Learning Disabilit*” OR “Mental handicap” OR “mental retardation” OR “intellectual impairment” OR “mentally disabled person” OR “learning disorder” OR “developmental disorder” )) AND (( parent* OR carer OR caregiver OR sibling OR famil* OR father OR mother OR sibling* OR brother* OR sister* OR guardian OR staff OR “service staff” OR nurse OR doctor OR psycholog* OR teacher OR care assistant OR “respite worker” OR “care worker” OR “Direct Support Professional” OR “Direct Care Staff” OR “social worker” OR “multidisciplinary team” OR "multi-disciplinary team" OR keyworker OR “person support worker” OR “direct support” )) AND (( “romance” OR “romantic relationship” OR sexual* OR “sexual consent” OR “sexual relationship” OR “couple” OR “love” OR “intimate” OR “intimacy” OR “significant other” OR “dating” OR “cybersex” OR “pornography” OR erection OR orgasm* OR masturbat* OR dating OR prostitute )) AND (( communicat* OR “interpersonal communication” OR discuss* OR narrat* OR talk OR convers* OR chat OR dialogue OR ("Conversation") OR consult* OR exchange OR education OR “discourse” OR “Negotiat*” OR “voice” OR “discussion” OR “social” OR “contact” OR “share” OR “response” OR “disclose” OR “text” OR “educat*” OR “interaction” OR “information” OR “guidance” OR “help” OR “address” OR "ask"))

**CINAHL:**

( Intellectual Disabilit* OR "Intellectual Development Disorder" OR "Down's Syndrome" OR “Downs Syndrome”OR “Developmental Disabilit*” OR “Learning Disabilit*” OR “Mental handicap” OR “mental retardation” OR “intellectual impairment” OR “mentally disabled person” OR “learning disorder” OR “developmental disorder” ) AND ( parent* OR carer OR caregiver OR sibling OR famil* OR father OR mother OR sibling* OR brother* OR sister* OR guardian OR staff OR “service staff” OR nurse OR doctor OR psycholog* OR teacher OR care assistant OR “respite worker” OR “care worker” OR “Direct Support Professional” OR “Direct Care Staff” OR “social worker” OR “multidisciplinary team” OR keyworker OR “person support worker” OR “direct support” ) AND ( “romance” OR “romantic relationship” OR sexual* OR “sexual consent” OR “sexual relationship” OR “couple” OR “love” OR “intimate” OR “intimacy” OR “significant other” OR “dating” OR “cybersex” OR “pornography” OR erection OR orgasm* OR masturbat* OR dating OR prostitute ) AND ( communicat* OR “interpersonal communication” OR discuss* OR narrat* OR talk OR convers* OR chat OR dialogue OR (DE "Conversation") OR consult* OR exchange OR education OR “discourse” OR “Negotiat*” OR “voice” OR “discussion” OR “social” OR “contact” OR “share” OR “response” OR “disclose” OR “text” OR “educat*” OR “interaction” OR “information” OR “guidance” OR “help” OR “address” OR "ask")

**MEDLINE:**

(Intellectual Disabilit* or "Intellectual Development Disorder" or "Down's Syndrome" or "Downs Syndrome" OR "Developmental Disabilit*" or "Learning Disabilit*" or "Mental handicap" or "mental retardation" or "intellectual impairment" or "mentally disabled person" or "learning disorder" or "developmental disorder") AND (parent* or carer or caregiver or sibling or famil* or father or mother or sibling* or brother* or sister* or guardian or staff or "service staff" or nurse or doctor or psycholog* or teacher or care assistant or "respite worker" or "care worker" or "Direct Support Professional" or "Direct Care Staff" or "social worker" or "multidisciplinary team" or keyworker or "person support worker" or "direct support") AND ("romance" or "romantic relationship" or sexual* or "sexual consent" or "sexual relationship" or "couple" or "love" or "intimate" or "intimacy" or "significant other" or "dating" or "cybersex" or "pornography" or erection or orgasm* or masturbat* or dating or prostitute) AND (communicat* or "interpersonal communication" or discuss* or narrat* or talk or convers* or chat or dialogue or "Conversation" or consult* or exchange or education or "discourse" or "Negotiat*" or "voice" or "discussion" or "social" or "contact" or "share" or "response" or "disclose" or "text" or "educat*" or "interaction" or "information" or "guidance" or "help" or "address" or "ask")

**Scopus:**

TITLE-ABS-KEY ( intellectual AND disabilit* OR "Intellectual Development Disorder" OR de "Down's Syndrome" OR "Downs Syndrome" OR "Developmental Disabilit*" OR "Learning Disabilit*" OR "Mental handicap" OR "mental retardation" OR "intellectual impairment" OR "mentally disabled person" OR "learning disorder" OR "developmental disorder" ) AND ( parent* OR carer OR caregiver OR sibling OR famil* OR father OR mother OR sibling* OR brother* OR sister* OR guardian OR staff OR "service staff" OR nurse OR doctor OR psycholog* OR teacher OR care AND assistant OR "respite worker" OR "care worker" OR "Direct Support Professional" OR "Direct Care Staff" OR "social worker" OR "multidisciplinary team" OR keyworker OR "person support worker" OR "direct support" ) AND ( "romance" OR "romantic relationship" OR sexual* OR "sexual consent" OR "sexual relationship" OR "couple" OR "love" OR "intimate" OR "intimacy" OR "significant other" OR "dating" OR "cybersex" OR "pornography" OR erection OR orgasm* OR masturbat* OR dating OR prostitute ) AND ( communicat* OR "interpersonal communication" OR discuss* OR narrat* OR talk OR convers* OR chat OR dialogue OR ( de "Conversation" ) OR consult* OR exchange OR education OR "discourse" OR "Negotiat*" OR "voice" OR "discussion" OR "social" OR "contact" OR "share" OR "response" OR "disclose" OR "text" OR "educat*" OR "interaction" OR "information" OR "guidance" OR "help" OR "address" OR "ask" ) AND PUBYEAR > 2005 AND PUBYEAR < 2024
